# Colonic diverticular disease: a bibliometric and visual analysis of the top 50 cited publications

**DOI:** 10.1097/MS9.0000000000005036

**Published:** 2026-06-03

**Authors:** Raseel B. Almutairi, Batool Y. Altayeb, Ibrahim A. Alsinan, Yara A. Alorfi, Reema H. Almuneef, Taghred J. Aljohani, Abdulaziz A. Alhumam, Zaid A. Dajani, Abdullah E. Alsubhi, Khalifa A. Almulhim

**Affiliations:** a College of Medicine, Imam Abdulrahman Bin Faisal University, Dammam, Saudi Arabia; b College of Medicine, University of Jeddah, Jeddah, Saudi Arabia; c College of Medicine, Majmaah University, Al-Majmaah, Saudi Arabia; d College of Medicine, Taibah University, Medina, Saudi Arabia; e College of Medicine, King Faisal University, Al-Ahsa, Saudi Arabia; f Faculty of Medicine in Rabigh, King Abdulaziz University, Rabigh, Saudi Arabia; g Department of Surgery, King Fahad University Hospital, Imam Abdulrahman Bin Faisal University, Dammam, Saudi Arabia

**Keywords:** bibliometric analysis, citation trends, colonic diverticular disease, diverticulitis, diverticulosis, research impact

## Abstract

Colonic diverticular disease (CDD) is a prevalent gastrointestinal condition with evolving patterns in diagnosis, management, and research focus. However, no prior study has systematically mapped the academic literature shaping the field. This bibliometric and visual analysis aims to identify and evaluate the 50 most-cited publications on CDD. Articles were retrieved from the Web of Science Core Collection, and data were extracted on citation count, publication year, journal, country of origin, study design, level of evidence, authorship gender, and primary outcomes. Additionally, keyword co-occurrence and country collaboration networks were analyzed. Data were visualized using VOSviewer and Microsoft Excel. The most-cited articles were published between 1953 and 2020, with a concentration between 2010 and 2015. The United States accounted for 58% of the top-cited studies, with limited representation from low- and middle-income countries. Retrospective cohort studies, reviews, and guidelines were the most frequent study types, while level 5 and level 1 evidence predominated. Surgical management emerged as the most common thematic focus, with relatively few articles addressing patient-reported outcomes, microbiome-related mechanisms, or cost-effectiveness. A notable gender imbalance was observed, with male authors occupying most first and senior author positions. Citation performance varied widely, with Painter *et al* leading in total citations. This study highlights the dominant role of Western institutions and surgical perspectives in shaping the field and underscores underrepresented research domains. The findings may guide future research priorities and promote a more inclusive and balanced scholarly landscape in diverticular disease.

## Introduction

Diverticula are bulges that develop in the wall of the intestine and are a common structural change found in the human colon. In most people, colonic diverticulosis, which is the presence of these pouches in the colon, does not cause symptoms. However, about 25% of individuals will experience symptoms, a condition called colonic diverticular disease (CDD). The severity of this disease can vary, ranging from a mild symptomatic form without complications to more serious conditions involving acute inflammation or bleeding[1].

HIGHLIGHTS

- This study presents the first bibliometric and visual analysis of the top 50 most-cited publications in the field of colonic diverticular disease.
- The analysis shows that research has primarily focused on surgical management, with fewer studies addressing patient-centered outcomes, microbiome interactions, or cost-effectiveness.
- The United States dominates the research landscape in this field, with most top-cited studies originating from U.S.-based institutions and authors.
- A significant gender imbalance exists in authorship, with male first and senior authors vastly outnumbering their female counterparts.
- Thematic gaps and citation trends identified in this study offer guidance for future research priorities and highlight the need for greater diversity and global equity in scholarly contributions.

Diverticular disease of the colon is a leading cause of hospital admissions and significantly drives up healthcare costs in Western and developed countries. These clinical and financial challenges highlight its increasing importance as a major public health issue^[^2,3^]^. In recent decades, global research on CDD has expanded rapidly, reflecting increased interest in its epidemiology, pathogenesis, and evolving management strategies. Key advancements include refining diagnostic criteria, the development of antibiotic-sparing approaches, and a greater focus on personalized treatment decisions in both medical and surgical care[4]. Despite this expanding body of research, the field remains methodologically varied and somewhat fragmented, with inconsistent outcome measures, different levels of evidence, and diverse study designs.

There is no comprehensive analysis that identifies the most influential publications in this field. While individual subtopics have been reviewed, a consolidated, citation-based synthesis is needed to highlight foundational studies, map paradigm shifts, and set future research priorities. Bibliometric analyses provide structured insights into a field’s literature, tracking authorship patterns, citation impact, and the development of knowledge over time^[^5,6^]^. Although hundreds of articles on diverticular disease are published each year, a thorough bibliometric analysis of CDD is still lacking. In contrast, similar analyses have been successfully applied to other gastrointestinal diseases, such as colorectal cancer (CRC) and inflammatory bowel disease (IBD)^[^7,8^]^.

This study aimed to perform a comprehensive bibliometric and visual analysis of the 50 most-cited publications on CDD. The main objectives were to identify and characterize these influential studies based on publication trends, study design, citation metrics, and levels of evidence. It also focused on analyzing authorship patterns, institutional affiliations, and geographic contributions. Secondary objectives included mapping thematic research trends and keyword networks to reveal emerging areas of interest and knowledge gaps within the field. Additionally, the study sought to assess gender representation among first and senior authors to highlight potential disparities in authorship within highly cited literature on CDD. This study adheres to the TITAN (Transparency in the Reporting of Artificial Intelligence) 2025 reporting guidelines[9]. No artificial intelligence tools were used in the design, analysis, or writing of this manuscript.

## Methodology

### Study design and search strategy

This bibliometric and visual analysis was conducted to identify and characterize the 50 most-cited publications on CDD. The method of selecting only the top 50 most-cited articles was applied to facilitate a focused and detailed analysis, a practice employed in multiple previous bibliometric analysis studies. Focusing on the top 50 most-cited papers enabled identification of the most influential and foundational contributions in the field while preserving the readability and interpretability of bibliometric maps. The Web of Science Core Collection (WoSCC) was selected as the primary database due to its wide coverage of peer-reviewed journals and reliable citation metrics. No date or publication type restrictions were applied to ensure the inclusion of both foundational and recent influential works.

A comprehensive search was performed on 2 March 2025. The search strategy was (“Diverticular Diseases” OR “Diverticular Disease” OR “Diverticular Bleeding” OR “Diverticular Bleedings” OR “Diverticulum” OR “Diverticula” OR “Diverticulosis” OR “Diverticulitis” OR “Diverticulitides”), with no special filters. The search yielded 30 898 results, which were arranged using the “time Cited highest to lowest” link on the Web of Science (WoS) system. The results showed the articles organized in descending order, with the most frequently cited articles at the top. A copy of the results was downloaded to ensure no changes in the citation matrix.

### Screening and selection criteria

Two independent reviewers conducted a two-step screening process. First, article titles and abstracts were screened to exclude studies unrelated to CDD. Full texts were then reviewed by two additional reviewers to confirm relevance, and discrepancies were resolved by a third reviewer. Records were eligible for inclusion if they focused on CDD, involved human subjects or patient populations, were published in English, and ranked among the top 50 most-cited publications in the field. Studies were excluded if they did not primarily address CDD, did not involve human subjects or patient populations, were not published in English, or were not among the top 50 cited records. The restriction to English-language publications was applied to ensure consistency and reliability in text-based bibliometric analyses and because English represents the predominant language of publication in this field. Duplicate records were checked manually, and any duplicates identified within the initial top 50 were removed immediately. Self-citations were not adjusted for, as citation-based indicators were used descriptively to reflect the overall structure and impact of the literature, consistent with common bibliometric practice. Moreover, inter-reviewer agreement during the study selection process was assessed using Cohen’s kappa coefficient. Cohen’s kappa demonstrated substantial agreement between reviewers during both phases of study selection, with a kappa value of 0.75 for the title and abstract screening phase and 0.76 for the full-text eligibility assessment. The final selection comprised the 50 most highly cited articles, which were included in the full analysis. The methodology flowchart is provided in Supplemental Digital Content Material 1, available at: http://links.lww.com/MS9/B235.

### Data extraction and analysis

We used the bibliometrix R-package and its web interface, Biblioshiny, to perform core bibliometric analyses. We generated multiple plots and tables to illustrate key bibliometric indicators, including a time-series plot of scientific production growth, authors’ production over time, the most relevant authors and journals, the most-cited documents, and keyword co-occurrence networks. In addition to automated bibliometric outputs, several variables were extracted manually by reviewing the full text of each article. These included study design, level of evidence (based on the Oxford Centre for Evidence-Based Medicine), primary outcome, and the gender of the first and senior authors (determined via institutional or professional profiles). We also used Microsoft Excel as a complementary tool to organize, clean, and visualize data. Excel was employed to tabulate extracted variables (e.g., study design, citation metrics), calculate normalized citation indices, and generate custom bar charts, pie charts, and summary tables not directly available through Biblioshiny. These visual elements were used to supplement the analysis and improve interpretability. Figures generated in Excel were reviewed and cross-referenced with raw data to ensure accuracy.

## Results

### Characteristics of the included studies

**Figure 1.**
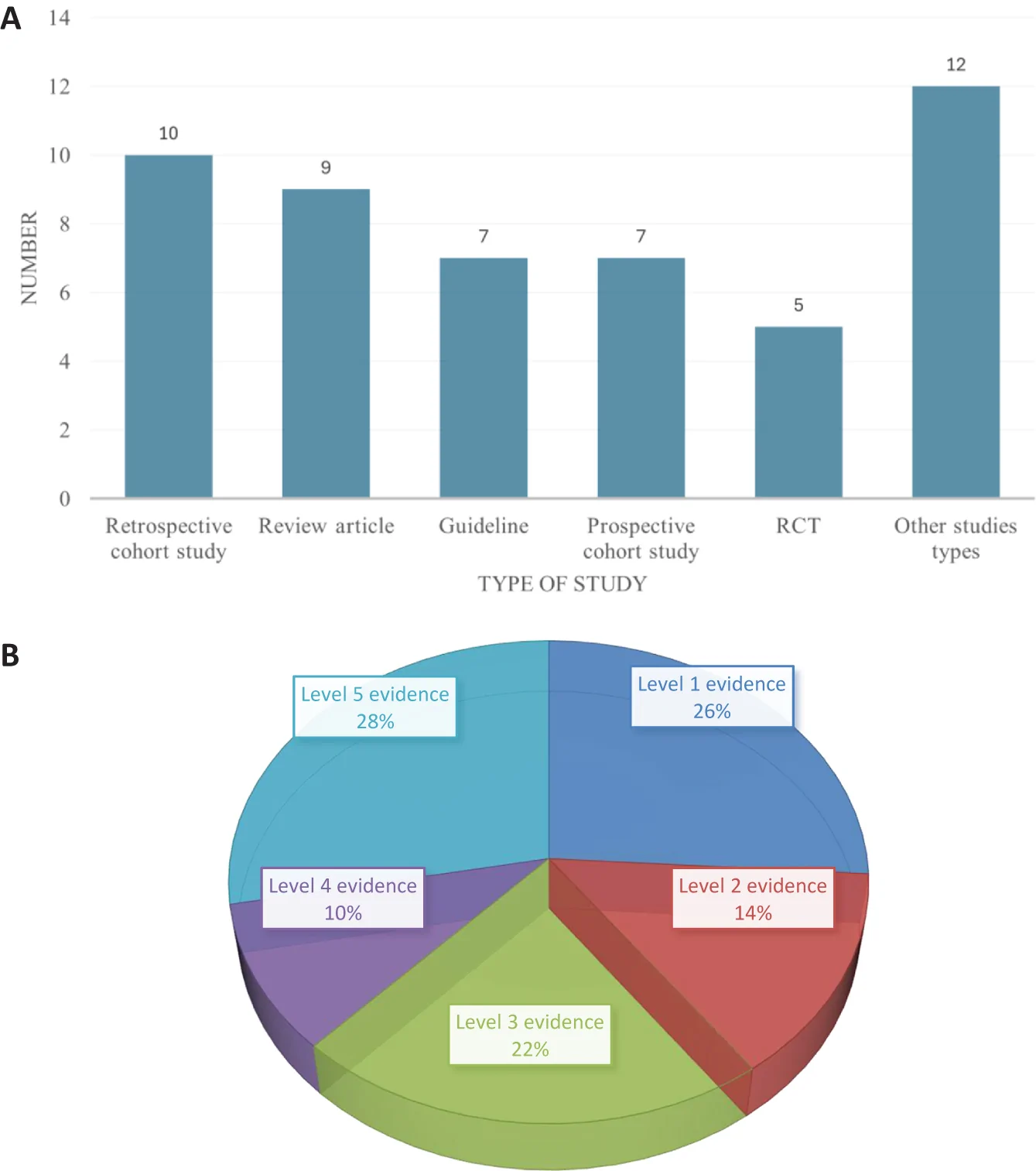
(A) Bar chart demonstrating the various types of the top 50 most-cited studies in diverticular disease research; (B) Pie chart illustrating the levels of evidence among the top 50 most-cited studies in diverticular disease research.

Table 1 presents the most-cited papers in the field of diverticular disease, including total citation (TC) counts, citations per year, and normalized citation index. The most highly cited article, by Painter *et al* (1971), published in the *British Medical Journal*, has accumulated 722 TCs, averaging 13.13 citations per year. Among the top 50 most influential studies in the field, the included studies encompassed a broad range of research designs, reflecting methodological diversity. The most common study type was the retrospective cohort study (*n* = 10), followed by review articles (*n* = 9), guidelines (*n* = 7), prospective cohort studies (*n* = 7), and randomized controlled trials (RCTs) (*n* = 5). Other study types included experimental physiological studies, epidemiological articles, physiological studies, radiological comparison studies, descriptive studies, cross-sectional studies, consensus development conferences, retrospective reviews, systematic reviews, ecological hypothesis-driven observational studies, clinical practice reviews, and observational postmortem studies – each represented by a single publication (Fig. 1A). In terms of quality, the levels of evidence varied considerably (Fig. 1B). Level 5 evidence (typically representing expert opinion or review articles) was the most common, accounting for 14 studies. This was closely followed by Level 1 evidence (e.g., RCTs and systematic reviews), present in 13 studies. Level 3 evidence comprised 11 studies. Level 2 evidence (e.g., prospective cohort studies) accounted for seven studies, while Level 4 (e.g., cross-sectional or retrospective cohort studies) included five studies. Full characteristics of each included study are detailed in Supplemental Digital Content Material 2, available at: http://links.lww.com/MS9/B236.

**Table 1:** The citation parameters of the top 50 most-cited studies in diverticular disease research.

Paper	Total citations	TC per year	Normalized TC^a^
Painter NS, 1971, *BMJ* – *British Medical Journal*^[^10^]^	722	13.13	1.00
Parks TG, 1975, *Clinics in Gastroenterology*^[^11^]^	526	10.31	1.00
Rafferty J, 2006, *Dis Colon Rectum*^[^12^]^	533	26.65	1.00
Jensen DM, 2000, *N Engl J Med*^[^13^]^	482	18.54	1.17
Stollman N, 2004, *Lancet*^[^14^]^	486	22.09	1.28
Etzioni Da, 2009, *Ann Surg*^[^15^]^	482	28.35	1.41
Feingold D, 2014, *Dis Colon Rectum*^[^16^]^	421	35.08	1.00
Parks TG, 1969, *BMJ*^[^17^]^	391	6.86	1.09
Köhler L, 1999, *Surg Endosc-Ultrason Interv Tech*^[^18^]^	385	14.26	1.30
Kaiser AM, 2005, *Am J Gastroenterol*^[^19^]^	390	18.57	1.46
Chabok A, 2012, *Br J Surg*^[^20^]^	385	27.50	1.43
Wong WD, 2000, *Dis Colon Rectum*^[^21^]^	342	13.15	0.83
Jacobs DO, 2007, *N Engl J Med*^[^22^]^	338	17.79	1.00
Hughes LE, 1969, *Gut*^[^23^]^	326	5.72	0.91
Shahedi K, 2013, *Clin Gastroenterol Hepatol*^[^24^]^	320	24.62	1.00
Stollman NH, 1999, *Am J Gastroenterol*^[^25^]^	300	11.11	1.01
Strate LL, 2009, *Gastroenterology*^[^26^]^	287	16.88	0.84
Oberkofler CE, 2012, *Ann Surg*^[^27^]^	275	19.64	1.02
Salem L, 2004, *Dis Colon Rectum*^[^28^]^	271	12.32	0.72
Hall J, 2020, *Dis Colon Rectum*^[^29^]^	263	43.83	1.12
Almy TP, 1980, *N Engl J Med*^[^30^]^	262	5.70	1.00
Myers E, 2008, *Br J Surg*^[^31^]^	274	15.22	1.00
Klarenbeek BR, 2009, *Ann Surg*^[^32^]^	259	15.24	0.76
Strate LL, 2012, *Am J Gastroenterol*^[^33^]^	262	18.71	0.97
Strate LL, 2019, *Gastroenterology*^[^34^]^	255	36.43	1.00
Ferzoco LB, 1998, *N Engl J Med*^[^35^]^	251	8.96	1.04
Chapman J, 2005, *Ann Surg*^[^36^]^	255	12.14	0.95
Janes S, 2005, *Br J Surg*^[^37^]^	242	11.52	0.90
McGuire HH, 1994, *Ann Surg*^[^38^]^	241	7.53	1.03
Krukowski ZH, 1984, *Br J Surg*^[^39^]^	233	5.55	1.06
Bharucha AE, 2015, *Am J Gastroenterol*^[^40^]^	230	20.91	1.04
Morson BC, 1963, *Br J Radiol*^[^41^]^	226	3.59	1.00
Daniels L, 2017, *Br J Surg*^[^42^]^	228	25.33	1.00
Gear JSS, 1979, *Lancet*^[^43^]^	227	4.83	1.00
Anaya DA, 2005, *Arch Surg*^[^44^]^	235	11.19	0.88
Stollman N, 2015, *Gastroenterology*^[^45^]^	230	20.91	1.04
Aldoori WH, 1994, *Am J Clin Nutr*^[^46^]^	226	7.06	0.97
Peery AF, 2012, *Gastroenterology*^[^47^]^	225	16.07	0.83
Hulnick DH, 1984, *Radiology*^[^48^]^	206	4.90	0.94
Painter NS, 1964, *Gut*^[^49^]^	206	3.32	1.00
Broderick-Villa G, 2005, *Arch Surg*^[^50^]^	217	10.33	0.81
Sartelli M, 2020, *World J Emerg Surg*^[^51^]^	206	34.33	0.88
Vennix S, 2015, *Lancet*^[^52^]^	205	18.64	0.92
Aldoori WH, 1998, *J Nutr*^[^53^]^	230	8.21	0.96
Ambrosetti P, 2002, *Eur Radiol*^[^54^]^	201	8.38	1.00
Andersen JC, 2012, *Dan Med J*^[^55^]^	201	14.36	0.75
Weizman AV, 2011, *Can J Gastroenterol Hepatol*^[^56^]^	204	13.60	1.00
Wasvary H, 1999, *Am Surg*^[^57^]^	202	7.48	0.68
Painter NS, 1965, *Gastroenterology*^[^58^]^	190	3.11	1.00
Welch CE, 1953, *Ann Surg*^[^59^]^	186	2.55	1.00

^a^ Normalized TC was calculated as the ratio between the total citations of a paper and the average total citations of all papers published in the same year.

### The most relevant authors and production over time in the research of diverticular disease

**Figure 2.**
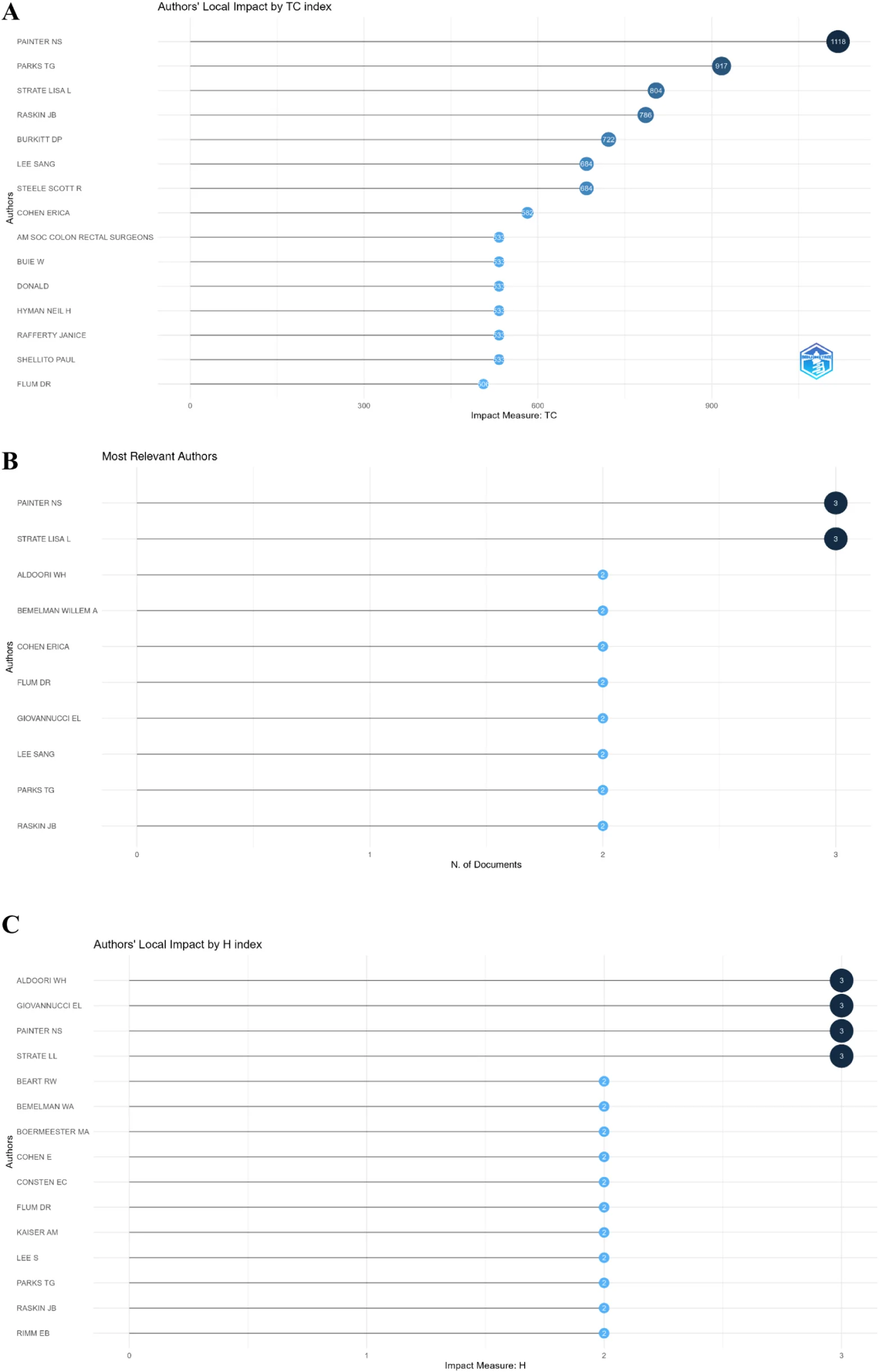
(A) Author impact based on citations; (B) Most relevant authors based on the number of articles; (C) Author impact based on the *h*-index.

*Painter NS* is the author with the highest impact, having a TC count of 1118, as shown in Fig. 2A, followed by *Parks TG* with a TC of 917 and *Strate Lisa L* with a TC of 804. Other notable contributors include *Raskin JB* (786) and *Burkitt DP* (722). This distribution indicates that these authors have made significant contributions to the field, as reflected by the high number of citations their work has received. Figure 2B quantifies the contributions of researchers in the field of diverticular disease among the top 50 publications by the number of documents they have published. *Painter NS* and *Strate Lisa L* are the leading contributors, each with three published articles. The other authors have each contributed two articles to the body of research. Figure 2C illustrates the local impact of selected authors, measured by their *h*-index. Four authors (*Aldoori WH, Giovannucci EL, Painter NS*, and *Strate LL*) show the highest local impact with an *h*-index of 3, indicating a comparatively stronger and more consistent citation performance in this research corpus. The remaining authors cluster at an *h*-index of 2, reflecting a moderate but still meaningful contribution to the field.

### Authorship gender distribution

The analysis of the top 50 most-cited studies in diverticular disease research revealed a notable gender imbalance among authors. Of these studies, 6 were single-author articles written by males, while 31 articles featured both a male first author and a male senior author. Additionally, 10 studies had a female first author with a male senior author, and 1 study had a male first author with a female senior author. Furthermore, we found that in two studies, the first and senior author was the same individual, one study being a female and the other being a male. These findings highlight a predominance of male authorship, particularly in senior authorship positions within the field.

### Publication trends and journal impact in diverticular disease research

**Figure 3.**
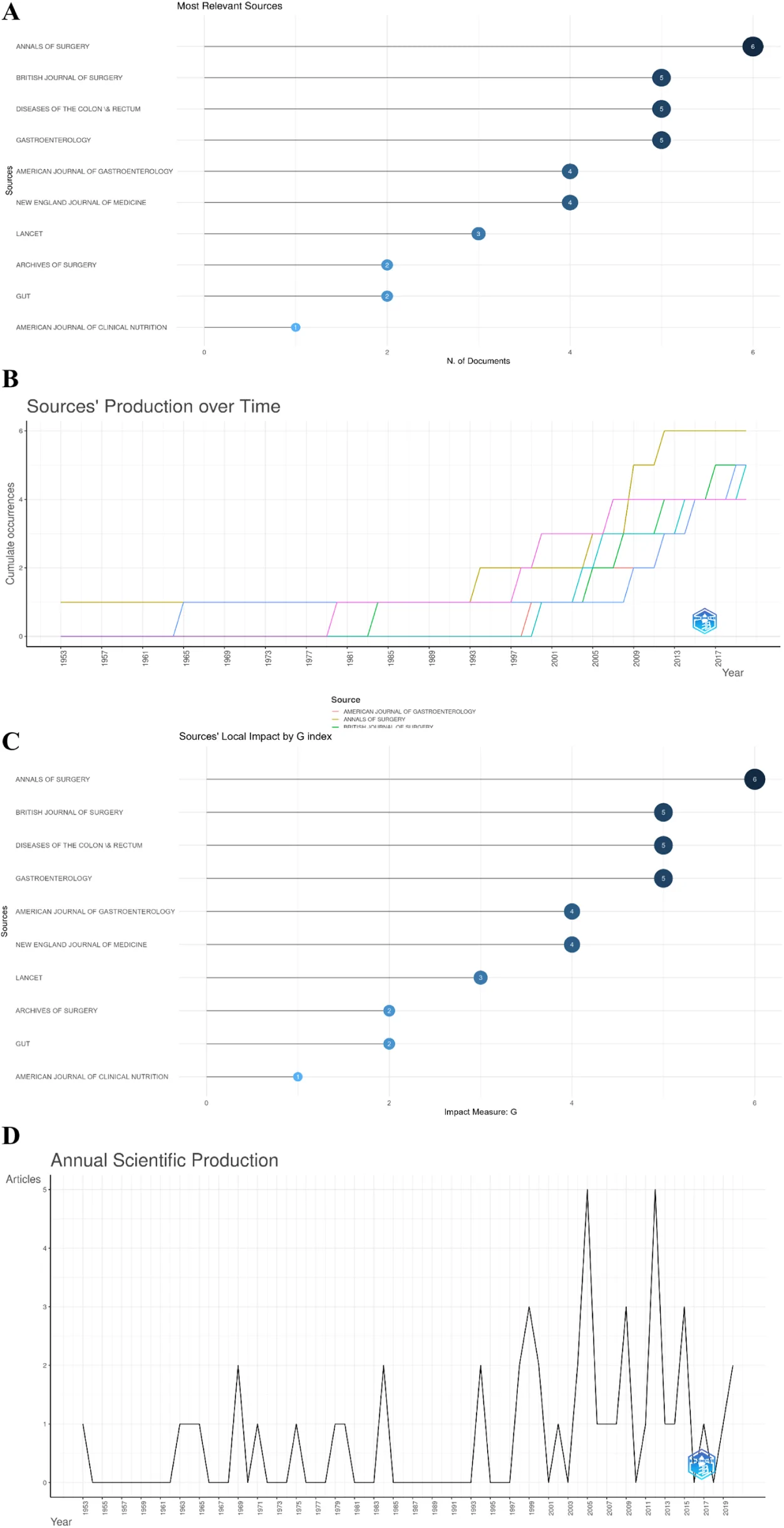
(A) Publication trends of the most productive journals among the top 50 most-cited studies in diverticular disease research; (B) Journal publication trends over time among the top 50 most-cited studies in diverticular disease research; (C) Sources’ local impact based on *g*-index; (D) Time-series plot showing the publication growth trend among the top 50 most-cited studies in diverticular disease research.

The most relevant sources where the top 50 articles on diverticular disease were published are presented in Fig. 3A. The figure depicts the 10 most productive journals, with *Annals of Surgery* leading the way, having published six articles. This is followed by *British Journal of Surgery, Gastroenterology*, and *Diseases of the Colon and Rectum*, each with five articles. Other notable journals include *American Journal of Gastroenterology* and *New England Journal of Medicine*, each contributing four publications. This data highlights *Annals of Surgery* as the most productive and relevant journal in the field of diverticular disease research. Figure 3B shows the cumulative number of publications over time across six journals. Overall, publication output increased gradually, with most growth occurring after 1990. *Annals of Surgery* had the highest cumulative number of publications, reaching six by the mid-2010s. *British Journal of Surgery, Diseases of the Colon & Rectum*, and *Gastroenterology* demonstrated moderate increases beginning in the early 2000s. *New England Journal of Medicine* showed earlier but limited growth, while the *American Journal of Gastroenterology* had the most recent onset of publications. Figure 3C represents the local impact of source journals based on the *g*-index within the analyzed literature. *Annals of Surgery* shows the highest local influence (*g*-index = 6), indicating both high productivity and strong citation performance in this dataset. A second tier of influential sources includes *British Journal of Surgery, Diseases of the Colon & Rectum*, and *Gastroenterology* (*g*-index = 5), highlighting their central role in disseminating impactful research. High-prestige multidisciplinary journals such as the *American Journal of Gastroenterology* and *New England Journal of Medicine* also demonstrate substantial local impact (*g*-index = 4). Figure 3D depicts the annual scientific production of research related to diverticular disease from 1953 to 2020. The graph reveals fluctuations in research output over the years, with a notable increase in activity starting in the early 2000s. This upward trend reflects growing interest and awareness of diverticular disease as a significant health concern. Periods of heightened academic focus are evident from the peaks in publication numbers, indicating an evolving understanding of the disease’s causes, risk factors, and treatment options. This trend is particularly pronounced in countries with advanced healthcare systems, where extensive literature reviews and systematic studies are more feasible. The graph underscores the increasing attention diverticular disease has garnered from researchers.

### Central countries and most relevant affiliations

**Figure 4.**
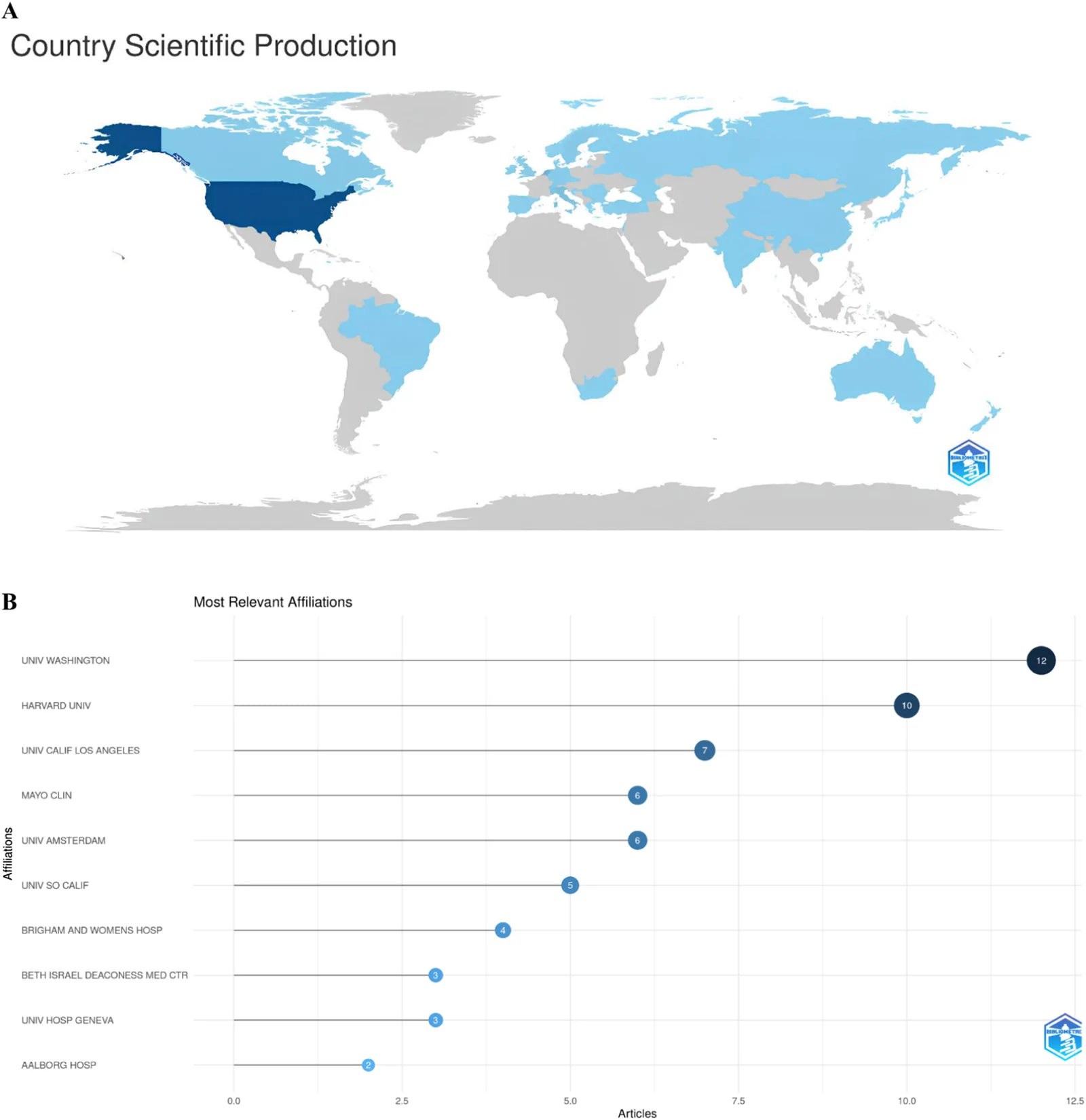
(A) Country’s scientific production among the top 50 most-cited studies in diverticular disease research; (B) Main research centers (affiliations) among the top 50 most-cited studies in diverticular disease research.

The world map shown in Fig. 4A highlights the countries that contribute the most to diverticular disease research. Among the top 50 most influential studies in the field of diverticular disease, the majority originated from high-income countries. The United States contributed the largest share, accounting for 58% of the publications, followed by the United Kingdom (16%), Switzerland, Italy, and the Netherlands (each contributing 4%). The remaining 14% came from Denmark, Canada, Ireland, Australia, New Zealand, Sweden, and Iceland. The darker the shade of blue on the map, the greater the intensity of scientific collaboration on the subject. Regarding institutional affiliations, the most prolific contributors are shown in Fig. 4B.

### Thematic areas and primary outcomes

The primary outcomes of the top 50 most-cited studies in diverticular disease research, detailed in Supplemental Digital Content Material 2, available at: http://links.lww.com/MS9/B236, were thematically categorized into seven major domains. The largest category was Surgical Management and Outcomes (*n* = 13), reflecting the central role of operative decision-making in the field. These included trials comparing Hartmann’s procedure with primary anastomosis^[^26,28^]^, laparoscopic versus open surgery^[^31,32,52^]^, and analyses of surgical timing, complications, and emergency interventions^[^36–39,44,57,59^]^. Some questioned the need for surgery altogether in select patients[50]. Epidemiology and Risk Factors (*n* = 11) was the second most prominent theme, including studies that investigated the disease’s natural history^[^11,15,17,24^]^, population-based incidence^[^10,23,33,40^]^, and associations with obesity, diet, or age^[^26,46,53^]^. Medical and Non-Surgical Management (*n* = 4) encompassed trials and observational studies examining conservative management strategies such as antibiotics, fiber intake, or observational treatment of uncomplicated diverticulitis^[^20,42,43,47^]^. Clinical Guidelines and Consensus Statements (*n* = 8) translated evidence into practice recommendations from major societies^[^12,16,21,25,29,45,51,55^]^. Diagnosis and Imaging (*n* = 5) evaluated the role of CT, colonoscopy, and radiologic comparisons in guiding management decisions^[^13,19,35,48,54^]^. Narrative Reviews and Scoping Articles (*n* = 6) offered comprehensive overviews of disease presentation, management, and chronicity^[^14,18,22,30,34,56^]^. Finally, Pathophysiology and Mechanistic Insights (*n* = 3) explored hypotheses related to colonic pressure, motility, and muscular abnormalities^[^41,49,58^]^.

### Analysis of main keywords, thematic evolution, and thematic map

**Figure 5.**
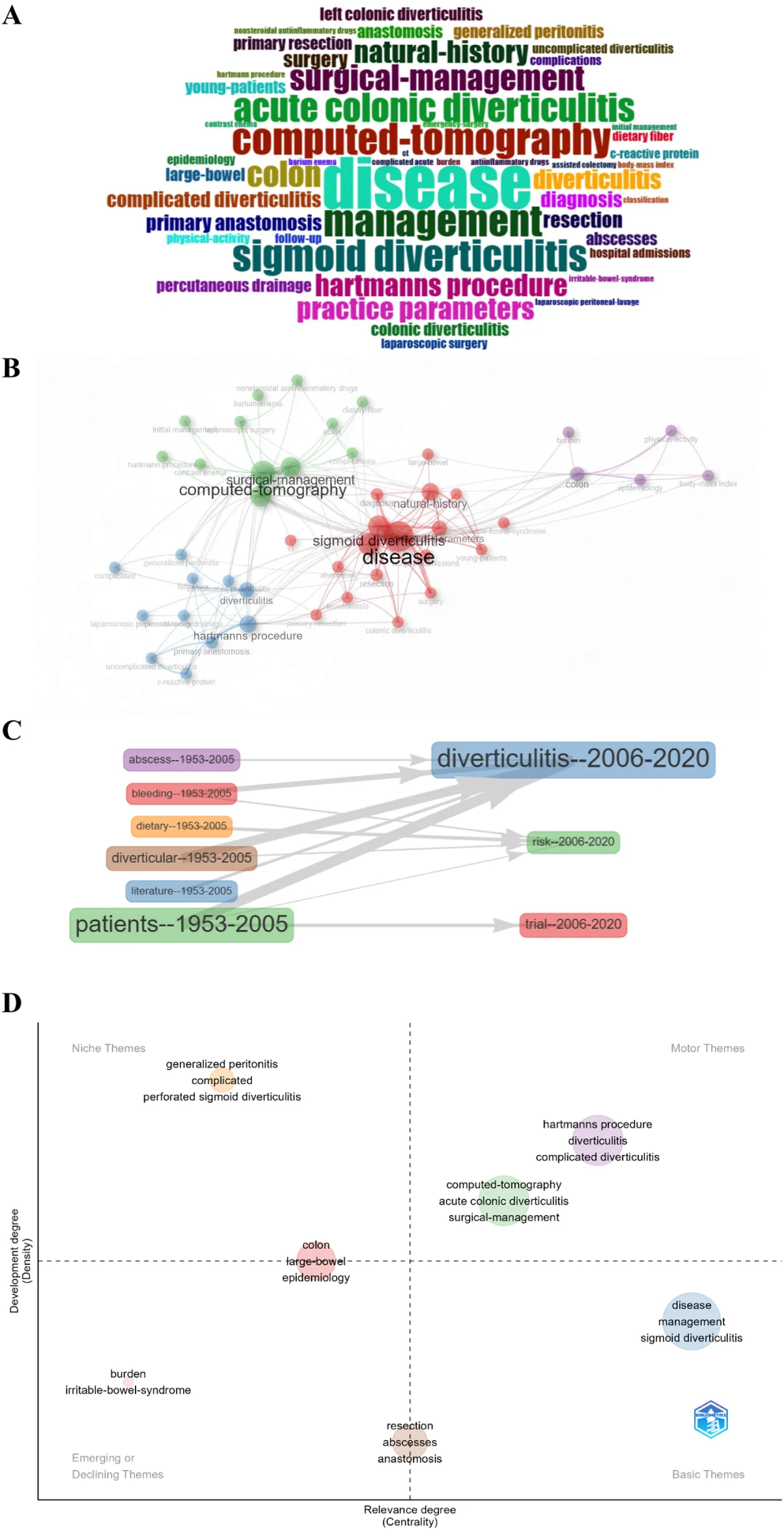
(A) Word cloud representing the most frequently occurring keywords in the literature on diverticular disease. The size of each word reflects its frequency, with larger words indicating more frequent appearances across the dataset; (B) Co-occurrence network of keywords illustrating thematic clusters and interconnections based on their frequency and co-occurrence in diverticular disease literature; (C) Thematic evolution over time in the literature on diverticular disease; (D) Thematic map in diverticular disease literature.

The word cloud and co-occurrence network, as demonstrated in Fig. 5, offer valuable insights into the structure of research on diverticular disease. The word cloud (Fig. 5A) highlights the most frequently occurring terms, such as “disease,” “diverticulitis,” and “sigmoid diverticulitis,” reflecting key areas of focus. The co-occurrence network (Fig. 5B) further visualizes how these keywords are interconnected, with larger nodes representing more influential terms and thicker lines indicating stronger associations. Central nodes like “disease,” “sigmoid diverticulitis,” and “computed tomography” demonstrate high relevance and connectivity across studies. Distinct clusters – colored for clarity – represent thematic groupings, such as diagnostic and natural history topics, surgical approaches, imaging, and treatment modalities.

The thematic evolution over time (Fig. 5C) shows a clear shift from early topics (1953–2005) focused on patients, diverticular disease, bleeding, abscesses, and dietary factors toward a more clinically refined emphasis in the later period (2006–2020), dominated by diverticulitis, risk assessment, and clinical trials. This transition reflects increasing attention to disease stratification, outcomes, and evidence-based management. Complementing this, the thematic map (Fig. 5D) highlights diverticulitis and its management as central motor and basic themes, indicating their high relevance and strong development within the field. Advanced surgical topics (e.g., Hartmann’s procedure, complicated diverticulitis) appear as motor themes, while niche themes address severe complications such as perforation and generalized peritonitis.

### Co-citation network

**Figure 6.**
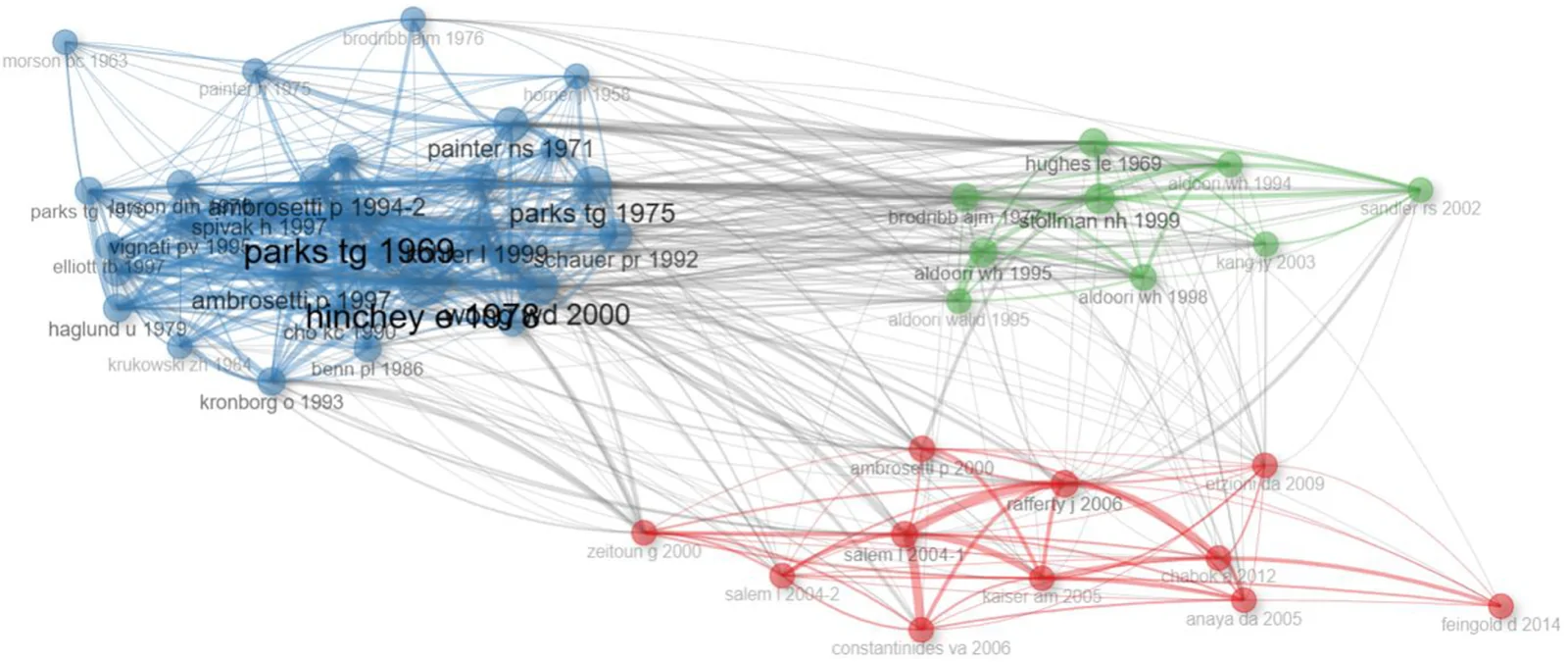
Co-citation network of references in the literature on diverticular disease.

This co-citation network (Fig. 6) illustrates the intellectual structure of the field by grouping frequently co-cited references into distinct clusters. Nodes represent individual publications, while links indicate co-citation relationships, with thicker connections reflecting stronger associations. The network reveals several well-defined clusters, suggesting distinct groups of closely related works, as well as substantial interconnections between clusters, indicating a shared and interconnected knowledge base. Larger and more centrally positioned nodes reflect highly influential references within the network.

## Discussion

This bibliometric analysis of the top 50 most-cited studies on CDD reveals a distinct intellectual landscape, primarily shaped by a small cluster of high-income countries, prominent institutions, and recurring authorship patterns. The most influential publications, both historically and currently, emphasize surgical management, the acute treatment of complications, and epidemiological risk profiling. This focus reflects the persistent clinical priority of managing severe disease and preventing recurrence, which collectively represent a significant source of morbidity and healthcare burden. Influential works, such as those by Rafferty *et al* and Wong *et al*, have long underscored the importance of surgical approaches.

### Citation trends and research activity

Our analysis of the citation performance among these highly influential publications indicates a clear evolutionary trend in the field. Between 2000 and 2015, the field experienced a notable surge in research output and citation impact. The period between 2010 and 2015 showed the highest concentration of influential papers and a peak in overall citation activity. This sustained increase reflects a period of heightened scientific inquiry and significant clinical advancements, pushing the understanding of CDD beyond mere observation.

### Geographic and institutional concentration

The geographic distribution of highly cited research is striking. The United States contributed the largest share, accounting for 58% of the top-cited studies, followed by the United Kingdom (16%), Switzerland, Italy, and the Netherlands (each contributing 4%). This concentration is likely a function of robust research infrastructure, greater access to high-impact journals, and broader visibility within international citation indices.

Crucially, there is a pronounced absence of highly cited research originating from low- and middle-income countries. This is a critical disparity, as variations in disease presentation, access to care, and treatment outcomes across diverse regions are currently underexplored in the leading literature. To address this imbalance, future efforts must focus on greater international collaboration, targeted regional capacity-building initiatives, and more inclusive publication practices that actively prioritize geographic diversity. It is important to mention that the geographic dominance of certain countries might reflect their relative representation in the WoS-indexed literature and may be influenced by database coverage, publication practices, and indexing policies, rather than solely by differences in research productivity.

### Methodological focus and evidence gaps

Methodologically, the most-cited studies predominantly include retrospective cohort studies, narrative reviews, and expert guidelines. While vital for generating hypotheses, synthesizing knowledge, and building clinical consensus, these designs are inherently susceptible to biases and often lack granular, patient-level outcomes. For instance, retrospective cohort studies can be limited by incomplete data, and narrative reviews do not employ the bias-minimizing methodologies of systematic reviews.

The goal of unifying clinical practice is further illustrated by the substantial number of highly cited review articles and guidelines. Although several RCTs were among the top 50 – such as those by Chabok *et al* and Daniels *et al*, which famously challenged the routine use of antibiotics in uncomplicated diverticulitis – high-level evidence from rigorously designed trials remains relatively sparse. Additionally, standardized outcome measures, patient-reported outcomes (PROs), and quality-of-life (QoL) data are less frequently emphasized in highly cited publications. This pattern reflects the thematic focus of the existing literature rather than the absence of research in these areas and should be interpreted in light of database coverage and citation practices.

### Underrepresented thematic research domains

Our comprehensive thematic analysis identified several significant, underexplored areas in the existing literature. Critical domains such as the intricate role of the gut microbiome, long-term patient QoL, cost-effectiveness of various interventions, shared decision-making models, and long-term functional outcomes remain notably underexplored within the most-cited publications. While surgical techniques and imaging strategies are discussed in depth, recurrence prevention strategies and the chronic psychosocial impact of CDD remain significantly understudied.

As CDD increasingly transitions from being viewed solely as an acute surgical emergency to a chronic gastrointestinal disorder requiring ongoing management, future research must critically broaden its scope. This expansion should prioritize patient-centered endpoints, investigate effective preventive strategies, and develop personalized management models that account for individual patient needs and disease trajectories.

### Comparative analysis with related fields

To contextualize the maturity and strategic gaps within CDD research, it is highly informative to compare its profile with bibliometric analyses of related gastroenterological conditions, such as CRC^[^8,60^]^ and IBD**^[^**7,61^]^.

The literature on IBD and CRC often demonstrates an earlier and stronger thematic focus on molecular pathogenesis and the intestinal microbiome in their foundational citations. In contrast, the top CDD literature shows a unique and persistent focus on surgical management, which is more pronounced than the thematic dominance seen in IBD. This disparity highlights a strategic gap: while IBD has integrated molecular and microbiome studies into its highest-impact literature, the CDD field has been slower to pivot toward these mechanistic insights, which are crucial for developing preventative and non-surgical therapies. The findings suggest that the scientific maturity of CDD, as measured by its most-cited works, lags behind that of IBD in adopting and establishing foundational research on molecular mechanisms. Furthermore, while all three conditions exhibit similar geographic concentration in Western nations, the methodological gaps identified in CDD – specifically the lack of highly cited papers on PROs, QoL, and economic burden – are particularly striking given the disease’s increasing prevalence and chronic impact. However, the prominence of surgical publications among the most-cited articles might reflect citation patterns within the WoS-indexed literature. This pattern may be influenced by the higher citation rates of surgical journals, the long-standing impact of foundational guidelines, and older landmark trials that continue to shape clinical practice.

### Gender imbalance in authorship

A key finding is the pronounced gender imbalance in authorship among the most highly cited works. The majority of both first and senior authors were male, reflecting broader systemic gender inequities prevalent in academic publishing and leadership positions within surgical and gastroenterological fields. This underrepresentation limits the diversity of perspectives and research questions and may subtly influence the prioritization of research areas. Addressing this requires intentional and sustained efforts, including targeted mentorship programs, inclusive authorship policies, and institutional accountability for diversity metrics. However, the observed differences in authorship by gender might reflect patterns within the indexed literature and should be interpreted as descriptive of publication representation rather than indicative of underlying causal factors.

### Limitations

This bibliometric analysis is subject to methodological limitations inherent to citation-based studies. First, this bibliometric analysis was conducted using the WoSCC as the sole data source. WoS was selected because it provides standardized, high-quality bibliographic records and reliable citation data, which are essential for robust bibliometric analyses. Although other databases, such as Scopus, Dimensions, and PubMed, offer broader coverage, especially for recently published or non-indexed journals, they were not included in order to avoid duplication, inconsistencies in citation counts, and differences in indexing policies across databases. Therefore, relevant publications not indexed in WoS may have been missed, which should be considered when interpreting the findings. Besides, the use of WoS may introduce coverage bias toward English-language, Western, and older high-impact journals, potentially underrepresenting research from other regions or newer sources. Moreover, the findings primarily reflect citation behavior within the WoS-indexed literature. Citation frequency may be influenced by factors such as publication age, visibility, database coverage, and the role of review articles and guidelines. Consequently, observed patterns should not be interpreted as direct indicators of research volume, methodological quality, or the intrinsic priority of specific topics within the field. Second, limiting the analysis to the top 50 most-cited articles creates a temporal bias, inherently favoring older, foundational publications that have had more time to accumulate citations over highly impactful recent works. Furthermore, citation count is an imperfect proxy for true scientific influence; it can be artificially inflated by factors like self-citation, author prominence, and journal visibility. Finally, the analysis of gender relies on publicly available information and only accounted for the first and senior authors, potentially underrepresenting the valuable contributions of co-authors and not accurately reflecting all individual gender identities.

### Future directions and implications

Within the limitations of a citation-based analysis, the findings highlight areas that appear relatively underrepresented in the highly cited literature on CDD. These patterns may inform discussions about potential avenues for future investigation, particularly in relation to PROs, QoL measures, and cost-effectiveness, which are less frequently emphasized among the most-cited studies despite their relevance to chronic disease management.

Similarly, the lower visibility of molecular and mechanistic studies, including research on the gut microbiome, reflects the thematic focus of the existing citation landscape rather than the absence of scientific activity in these areas. Future studies employing RCT designs and mechanistic approaches may help broaden the evidence base and complement the predominantly clinical and guideline-oriented literature.

Observed geographic and gender imbalances should also be interpreted as patterns within citation behavior and database coverage. These findings underscore the importance of ongoing efforts by research communities, institutions, and journals to promote inclusivity and diversity in scholarly publishing, rather than serving as prescriptive statements about research prioritization. Overall, these observations provide contextual insight into how the CDD literature has evolved and may help frame future discussions about balanced and comprehensive research development.

## Conclusion

This bibliometric review highlights both the depth and the structural disparities within the scientific literature on diverticular disease. It emphasizes the dominant influence of a limited group of authors, institutions, and countries in shaping the field, while drawing attention to persistent gaps in geographic representation and gender diversity. The sustained increase in publication volume since the early 2000s not only reflects growing academic interest but also signals the need for future research efforts that prioritize inclusivity and methodological innovation. These findings delineate critical patterns in academic output and offer a framework to guide future research priorities, enrich clinical education, and inform equitable policy development in the global management of CDD.

## Data Availability

All data generated or analyzed during this study are included in this manuscript and its supplementary files.

## Research registration unique identification number (UIN)

This study is a bibliometric analysis and does not involve human participants or data collected directly from individuals. Therefore, it does not require registration in a clinical trial or human studies registry.
